# 3D Printing of Customized Aspheric Lenses for Imaging

**DOI:** 10.3390/polym13203477

**Published:** 2021-10-10

**Authors:** Dexing Zhu, Jian Zhang, Qiao Xu, Yaguo Li

**Affiliations:** 1 Fine Optical Engineering Research Center, Chengdu 610041, China; starzhuxing@outlook.com; 2 Laser Fusion Research Center, China Academy of Engineering Research Center, Mianyang 621900, China; zhangjian.leo@163.com (J.Z.); xuqiao@vip.sina.com.cn (Q.X.)

**Keywords:** 3D printing, aspheric lenses, stereolithography, meniscus equilibrium method

## Abstract

A simple and efficient process for fabricating customized aspheric lenses is reported, in which a stereolithographic 3D printer combined with the meniscus equilibrium post-curing technique is employed. Two kinds of UV-curable resins, DentaClear and HEMA, were used for printing aspheric lenses in our experiments. The printed DentaClear lens featured low surface profile deviation of ~74 μm and showed satisfactory optical imaging resolution of 50.80 lp/mm, i.e., 4.92 μm. The surface roughness of the printed lens with DentaClear was measured to be around 2 nm with AFM. The surface roughness was improved as a result of post-curing, which reduced the ripples on printed lens surfaces. In contrast, the printed HEMA lens exhibited a significant stair-stepping effect with a large surface profile deviation of ~150 μm. The ripples were somewhat apparent even if the printed HEMA lens surface was smoothed by means of post-curing. No sharp image can be obtained with the HEMA lens in the resolution testing. The composition of HEMA resin may be the reason for the relatively poor surface quality and optical properties.

## 1. Introduction

Aspheric lenses are increasingly applied in various imaging systems to reduce spherical aberrations, distortion, and coma, as well as to correct pupil aberrations. Using aspheric surfaces instead of traditional spherical lenses can significantly reduce the number of optics in imaging systems [1]. The prevailing methods for producing aspheric lenses include lean manufacturing (injection molding, hot press molding, etc.) and subtractive manufacturing (e.g., computer-controlled polishing, ion beam figuring, magnetorheological finishing, etc.), which are generally time-consuming, inflexible, and cost-inefficient [2,3].

Three-dimensional (3D) printing, referring to the technology of fabricating 3D parts layer-by-layer from computer-aided design (CAD) models, is a promising method for fabricating elements that are challenging using traditional technologies, such as vascular stents [4], microfluidic devices [5], micro-lattices [6], and some optical components (for example, micro-lens arrays [7,8], ultracompact multi-lens objectives [9], and aspheric lenses [10,11,12]). Stereolithography (SLA) is a 3D printing technology that uses photocurable resin to manufacture objects [13], and characterizes high resolution and accuracy. In addition, besides polymers, ceramics and biocompatible materials can also be applied in SLA due to the development of photocurable composites [4,5]. Thus, SLA is widespread in aerospace, medicine, and optics. However, a major drawback in current 3D printing as well as SLA is the relatively poor surface quality resulting from the layer-by-layer method of the SLA process and the resultant “stair-stepping” effect [14]. To reduce the “stair-stepping” effect of printed optical components, some methods, including post-coating [15], meniscus equilibrium post-curing [16,17], and the grayscale photopolymerization method [18,19], have been proposed. The surface roughness of the smoothed parts can be reduced to a few nanometers with these methods. Consequently, there may be a potential process that combines 3D printing and surface smoothing to fabricate optical components efficiently and inexpensively.

In this paper, we report a simple and effective way to manufacture customized aspheric lenses with a stereolithographic 3D printer. Commercial UV-curable resin DentaClear and built-in-house resin HEMA (2-hydroxyethyl methacrylate) were utilized to print the designed aspheric lenses. The printed lenses were post-processed by meniscus equilibrium post-curing using the same resin, respectively. The printed lenses were characterized using optical microscope, laser confocal microscope, stylus profilometry, and atomic force microscopy (AFM). Furthermore, the imaging properties of the printed lenses were evaluated with the resolution chart.

## 2. Materials and Methods

### 2.1. Preparation of UV-Curable Resins

**Figure 1:**
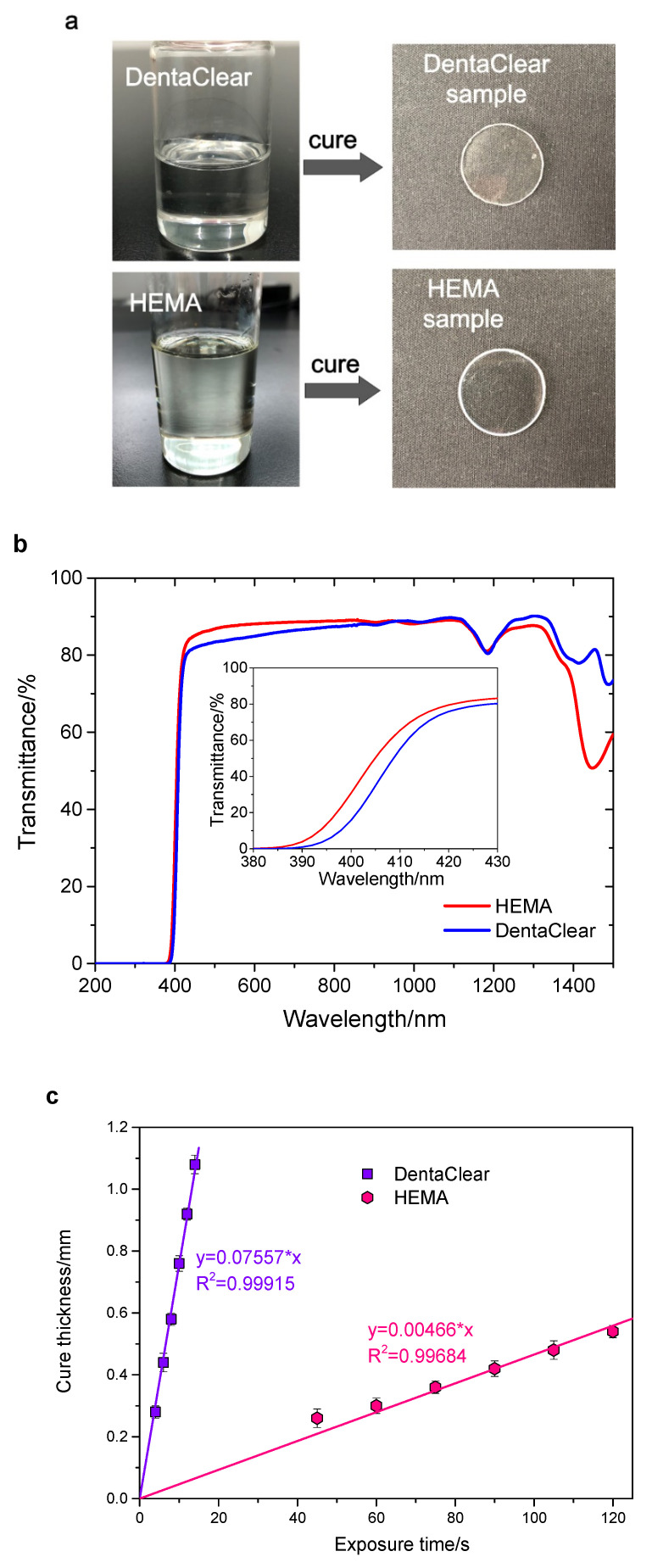
(**a**) Commercial UV-curable resin DentaClear, self-prepared resin HEMA, and test circle samples fabricated from DentaClear and HEMA. (**b**) Averages of spectral transmission curves measured on 5 samples. Both lenses made from DentaClear and HEMA possess high transparency in the visible range. (**c**) Curing thickness of the resins as a function of exposure time at a constant power of 32 mW/cm^2^. The curing thickness increases linearly with exposure time for both resins in the experimented time.

Two kinds of UV-curable resins, namely DentaClear and HEMA, were utilized to form aspheric lenses. As shown in Figure 1a, both resins appear transparent in the visible region. The commercial UV-curable resin DentaClear was purchased from JIE Technology Company Limited, Hong Kong. The self-prepared resin HEMA consisted of 98.6 wt.% HEMA (Aladdin, Shanghai, China, 99%) as a low-viscosity monomer, 1.0 wt.% diphenyl(2,4,6-trimethylbenzoyl) phosphine oxide (Aladdin, Shanghai, China) as a photo-initiator, 0.2 wt.% hydroquinone (Aladdin, Shanghai, China) as an inhibitor, and 0.2 wt.% UV327 (2,4-Di-tert-butyl-6-(5-chloro-2H-benzotriazol-2-yl) phenol) (purchased from Kangxin Co. Ltd, Beijing, China) as a UV-absorber.

The viscosity of DentaClear was measured to be 6.08 × 10^−1^ Pa·s at 0.5 rpm using a rotational viscometer (Brookfield LVDV II, Brookfield Engineering Laboratories, Middleboro, MA, USA), two orders of magnitude higher than HEMA (8.12 × 10^−3^ Pa·s at 50 rpm). The refractive indices of the cured parts from these two resins were similar, ~1.45 at the wavelength of 532 nm light, estimated using the total reflection method.

Ten samples with a diameter of 20 mm and a thickness of 1 mm (5 DentaClear samples and 5 HEMA samples) were tested for transmission in the spectral range of 200~1500 nm (Lambda 950, Perkin-Elmer, Boston, MA, USA). Figure 1a shows the appearance of DentaClear and HEMA prior to and following curing, and Figure 1b shows that spectral transmission averaged on 5 samples does not exhibit a significant difference for these two resins, DentaClear 87% and HEMA 88% on average, in between 430 and 1120 nm. Transmission of both samples sharply decreases near the UV edge of the spectra, owing to the addition of a UV-absorber in the resins (inset in Figure 1b).

The curing thickness of the resins with an exposure time at a constant power of 32 mW/cm^2^ is plotted in Figure 1c, which can be used to control the thickness of each cured layer. For both of the resins, the curing thickness is linearly proportional to the exposure time. The fitted linear equation of DentaClear is y=0.076x, while it is y=0.0047x for HEMA, indicating that HEMA is much more difficult to be cured compared to DentaClear.

### 2.2. Aspheric Lens Design

An aspheric lens was designed and optimized using commercial optical design software to reduce image distortion and spherical aberration. The aspheric surface profile can be expressed by:

(1) $$Z\left(r\right)=\frac{r^{2}}{R(1+\sqrt{1-\left(1+\kappa \right)\frac{r^{2}}{R^{2}}}}+A_{04}r^{4}+A_{06}r^{6}+\cdots$$

where *R* represents the radius of curvature, κ is the conic constant, and $A_{04}$, $A_{06}$, … are aspheric coefficients. The optical axis orientation is along the Z-direction, the diameter of the lens was 20 mm, and the focal length was designated to be 50 mm. In order to improve imaging resolution as well as to minimize field distortion, the surface profile of the lens was optimized at 532 nm light. The experimental refractive index of the cured samples was applied during the optimization process. As DentaClear and HEMA had a similar refractive index of 1.45 for green light, the optimized results were also similar.

**Figure 2:**
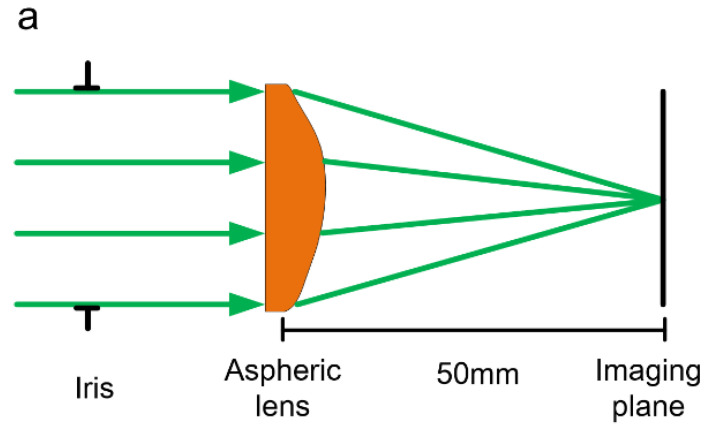

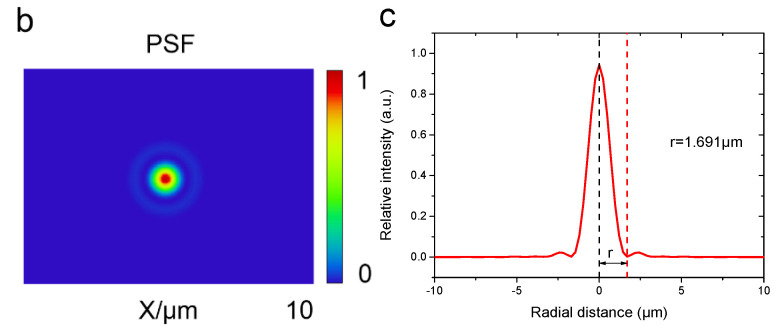
(**a**) Diagrammatic sketch of an optimal aspheric lens. (**b**) The 2D point spread function on the image plane. (**c**) The intensity profile of the point spread function of the designed lens.

The diagrammatic sketch of the optimized aspheric lens is shown in Figure 2a. The conic constant, κ, was set to be 0, and the optimized radius of the curvature, *R*, was calculated to be −22.62, which defined an axisymmetric quadric. The aspheric coefficients, *A_i_*, which represent the deviation of the surface from the axisymmetric quadric, were calculated to be *A*_04_ = 2.25 × 10^−5^ and *A*_06_ = 3.13 × 10^−9^. Imaging resolution of the optimized lens was analyzed in terms of the point spread function (PSF). In Figure 2b, the PSF takes the form of a circular shape with a central bright spot surrounded by gradually decreasing homocentric dark and bright rings. Generally speaking, the smaller and sharper the PSF is, the higher resolution the image system has. The distance from the center to the first dark ring is usually referred to as lateral resolution, $res=\frac{0.61\lambda }{NA}$ (according to the Rayleigh criterion), in which *λ* is the wavelength of light and *NA* is the numerical aperture of the lens. Based on the above-mentioned formula, the theoretical lateral imaging resolution of the designed aspheric lens is about 1.69 μm. 

### 2.3. Stereolithography Fabrication and Meniscus Equilibrium Post-Curing of Aspheric Lenses

**Figure 3:**
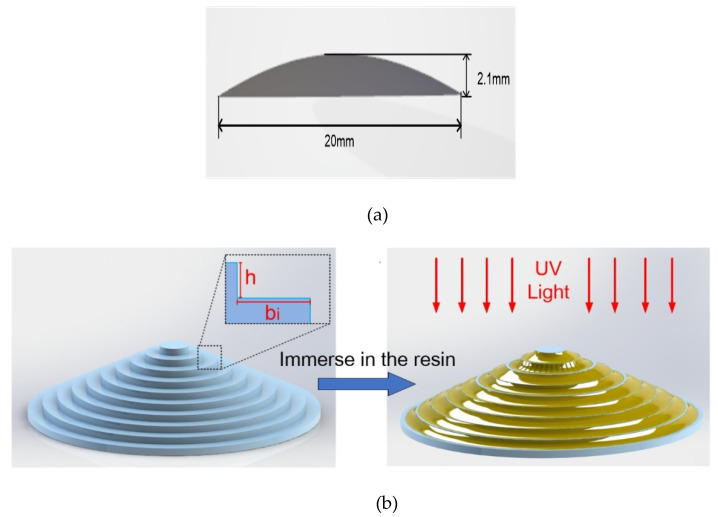

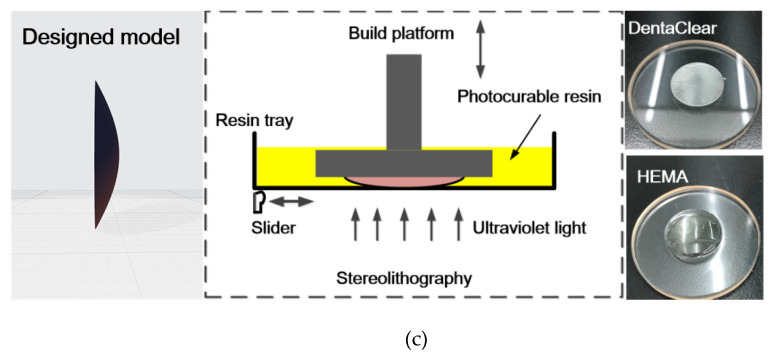
(**a**) STL model of the designed aspheric lens. (**b**) Sliced model of the designed lens and meniscus equilibrium post-curing process. (**c**) Two kinds of resins were structured in a stereolithography 3D printer respectively, and 3D-printed aspheric lenses on a fused silica substrate.

Prior to 3D printing of the designed lenses, a STL format model of the designed lens was created first, which approximates the designed lens with a grid of triangles (Figure 3a). The approximation parameters were an angle tolerance of 5° and a linear tolerance of 0.01 mm to guarantee the accuracy of the STL model [20]. The STL model was composed of 201,042 triangles. Then, the STL model was ready to be printed. After it is set up, the STL model is then sliced into multiple 2D layers with a preset layer thickness, *h*, while the width, *b_i_*, of each corresponding layer is calculated from the layer thickness, *h*, and the equation of the designed lens surface (Figure 3b). Then, each layer was solidified according to a mask image generated automatically by the printer. The printing of the designed lenses was completed after repeating the above steps until the laser layer was finished. The printed lenses need smoothing because they displayed the stair-stepping effect, which is inevitably incurred in the current 3D printing apparatus. It is well-known in fluid mechanics that a meniscus will be formed at the corner of stair steps in each layer when the printed lens with stair steps is immersed into and then emerges from liquid resins (Figure 3b). The liquid meniscus can be cured with UV light after being stabilized for a while. In this way, the printed lenses with stair steps can be smoothed by virtue of meniscus equilibrium post-curing. 

The SLA process was finished with a consumer-grade 3D printer (Pico2, Asiga, Alexandria, Australia). This printer has a bottom-up configuration, that is, the curing light with a wavelength of 385 nm is projected from underneath the resin tray to polymerize the resin. The building process is shown schematically in Figure 3c. First, the modeled lens to be printed was moved downwards into a tray containing UV-curable resin to the depth of one-layer thickness above the bottom of the tray. Then, a slider moved underneath the resin tray bottom made from a transparent Teflon film, lifting it level and squeezing out excessive photopolymer resin. Next, the mask image of the layer under construction was projected onto the underside of the tray film, causing resin to harden in the shape of the image. Then, the lens being printed was lifted out and separated from the tray film. This process was repeated until the whole lens was completely printed. In our experiments, the thickness of each layer was predetermined to be 10 μm and the designed model was sliced into 260 layers in total. During the course of printing the DentaClear lens, the light intensity of UV irradiation was 7.0 mW/cm^2^ and the exposure time was 5.7 s for curing each layer. On the other hand, the light intensity was elevated to 25 mW/cm^2^ and the exposure time to 15 s for HEMA. This stereolithography process took about 1~2 h for the whole stereolithography process, depending on the layer number and exposure time.

The printed lenses were immersed into the same resin from which they were composed after they were finished printing. Then, the lenses were lifted out of the resin to completely stabilize the liquid meniscus. Following that, the lenses were transferred into a UV-curing machine to solidify the meniscus. The lenses were finally cleaned in isopropanol solution with an ultrasonic cleaner so as to remove residual uncured resin. This post-curing process can be finished within 10 min. After the post-curing process, printed surfaces become much smoother.

## 3. Simulation of the Meniscus Equilibrium Post-Curing Process

**Figure 4:**
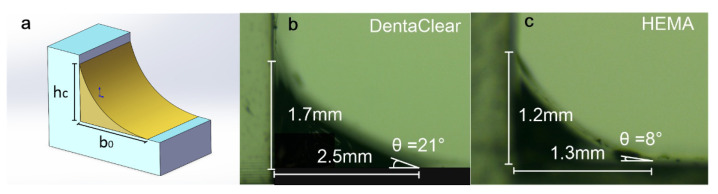

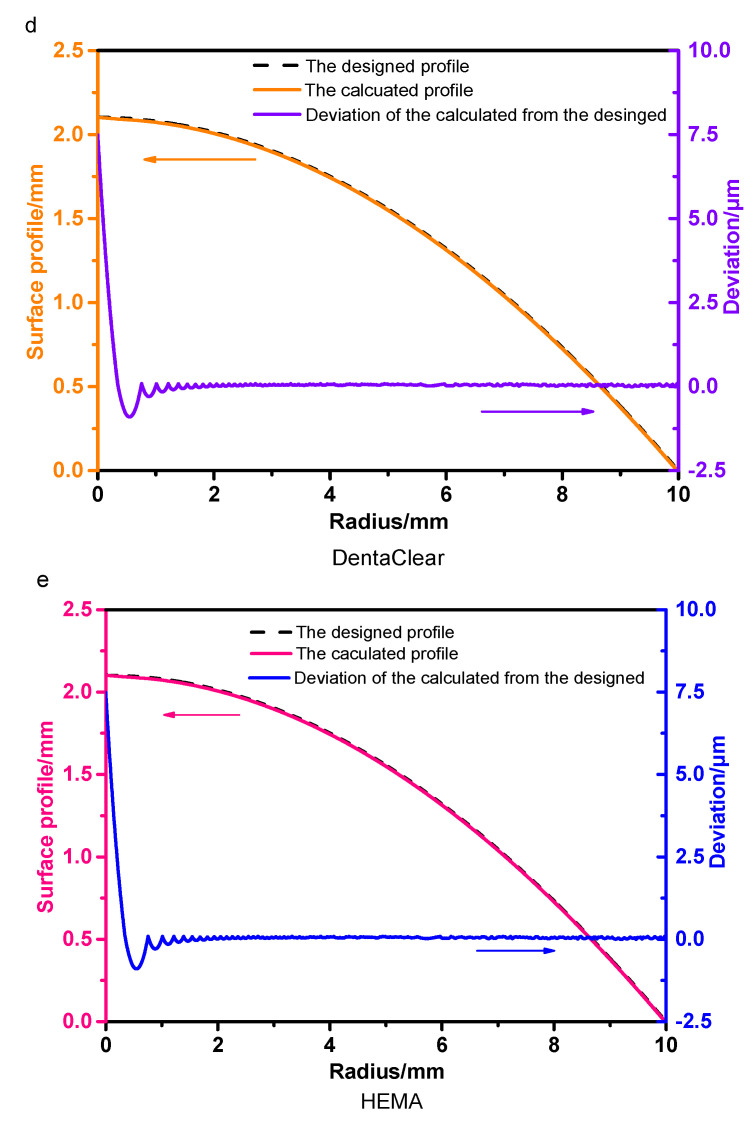
(**a**) Illustration of meniscus wetting to intersecting plane surfaces. (**b**) Built part for measuring the surface tension parameters of DentaClear. (**c**) Built part for measuring the surface tension parameters of HEMA. (**d**) Calculated surface profile and deviation of DentaClear lenses compared to the designed curve. (**e**) Calculated surface profile and deviation of HEMA lenses compared to the designed curve.

Theoretical simulation was performed to analyze the effectiveness of the meniscus equilibrium post-curing for surface smoothing. As described in [15,16,17], the developed meniscus shape equation relates the liquid interface to the influence of gravity and interfacial tension, which can be expressed by contact angle, θ, and capillary height, *h_c_*, in Equation (2):

(2) $$\rho gy-\frac{1}{2}h_{c}^{2}\times \frac{\rho g\ddot{y}}{(1-\sin\theta ){\left(1+{\dot{y}}^{2}\right)}^{\frac{2}{3}}}=0$$

where ρ is the liquid density, g is the gravitational acceleration, and y is the height of the meniscus above the horizontal plane. The capillary height, *h_c_*, in Equation (2) is the maximum height that the fluid can reach on an infinite vertical wall, as shown in Figure 4a. Similarly, the maximum length that the fluid can reach on an infinite horizontal plane is *b*_0_. The contact angle, θ, is the angle at which the liquid resin interface meets the cured resin surface.

For a given liquid–solid system, both parameters (*h_c_* and θ) can be experimentally measured. Figure 4b,c show the printed parts with horizontal and vertical planes used for measuring the contact angle, θ, and the capillary height, *h_c_*, of DentaClear and HEMA. It can be estimated that for DentaClear, θ = 21°, *h_c_* = 1.7 mm, and *b*_0_ = 2.5 mm. For HEMA, θ = 8°, *h_c_* = 1.2 mm, and *b*_0_ = 1.3 mm. In our experiments, the layer thickness during the stereolithography process was *h* = 0.01 mm. Based on the layer thickness and the equation of the designed lens surface, the width of the corresponding horizontal planes for each layer, *b_i_*, was computed, ranging from 0.0247 to around 0.3428 mm, where for both DentaClear and HEMA, *h* < *h_c_* and *b_i_* < *b*_0_. The boundary conditions of Equation (2) for all the layers would be:

(3) $$y\left(0\right)=h,\;y\left(b_{i}\right)=0$$

Then, the meniscus shape of each layer can be calculated by solving Equation (2) using MATLAB. The surface profiles of the smoothed lenses can be obtained by combining the solved meniscus curve of each layer. As the layer thickness, *h*, was much smaller than *h_c_*, the calculated profile of each layer is approximate to a straight line for both kinds of resin. Figure 4c,d compare the calculated profiles with the designed surfaces as well as the deviations of the calculated profiles from the designed DentaClear and HEMA lenses. The deviation between the calculated and the designed lenses ranged from −0.89 to around 7.48 μm for both resins. The deviation of the layers close to the top of the lens tends to be greater. According to the calculated results, meniscus equilibrium post-curing can smooth the lens surface with a deviation of less than 10 μm.

## 4. Results and Discussion

### 4.1. Surface Characterization of the Printed Lenses

**Figure 5:**
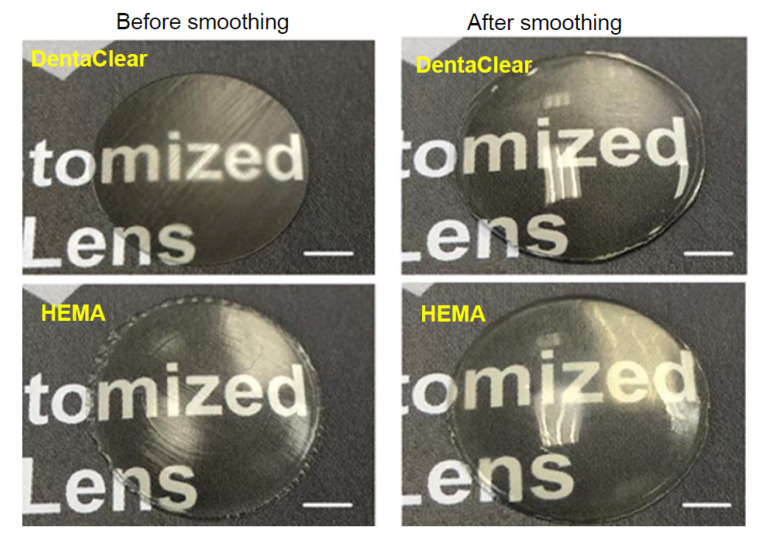
(**top**) Images of DentaClear lenses before (left) and after smoothing (right). (**bottom**) Images of HEMA lenses before (left) and after smoothing (right). Scale bars: 0.5 mm.

The surface properties of the printed lenses were characterized using optical microscope (OM), laser confocal scanning microscopy (LCSM), stylus profilometry, and atomic force microscopy (AFM). The printed lenses before and after the smoothing process were photographed (Figure 5). The lenses without smoothing show a significant stair-stepping effect and transverse scratches over the surface. The transverse scratches arise from the slider traces that remain on the resin tray film. In contrast, much smoother surface on lenses after smoothing were clearly demonstrated.

**Figure 6:**
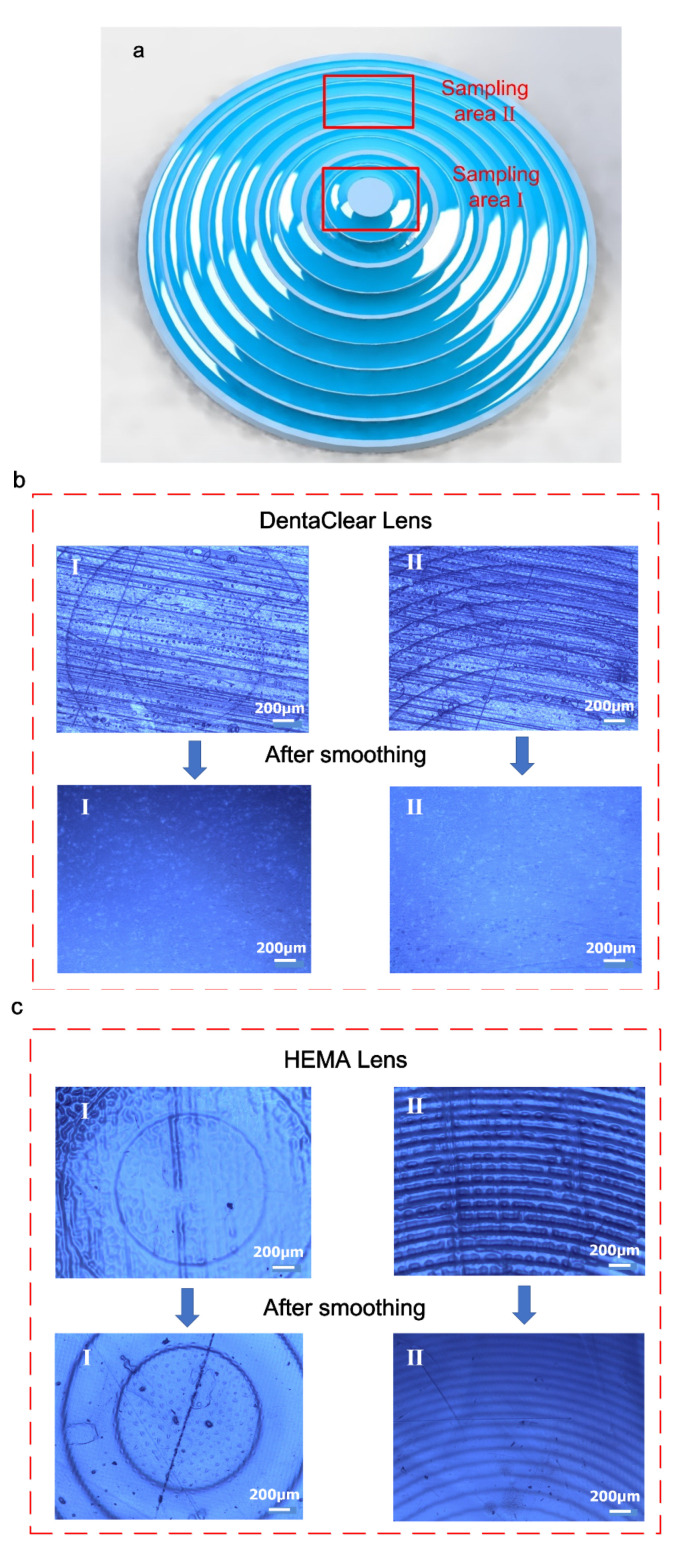
(**a**) Schematic diagram of the sampling areas under an optical microscope. (**b**) Optical microscope pictures of DentaClear lenses. (**c**) Optical microscope pictures of HEMA lenses.

The surface morphology was also observed with an optical microscope (Leica DMR, Wetzlar, Germany), as shown in Figure 6. Sampling area I is on the top region of the lens and sampling area II is on the side surface (Figure 6a). Both lenses fabricated from DentaClear and HEMA feature a serious stair-stepping effect and transverse scratches on the lenses before smoothing, while they possess smoother surfaces after post-curing. The DentaClear lens exhibited a much smoother surface on either the top or side surface, where the stair-stepping effect and transverse scratches could not be observed any more. For the HEMA lens, however, the stair-stepping effect was still perceptible.

**Figure 7:**
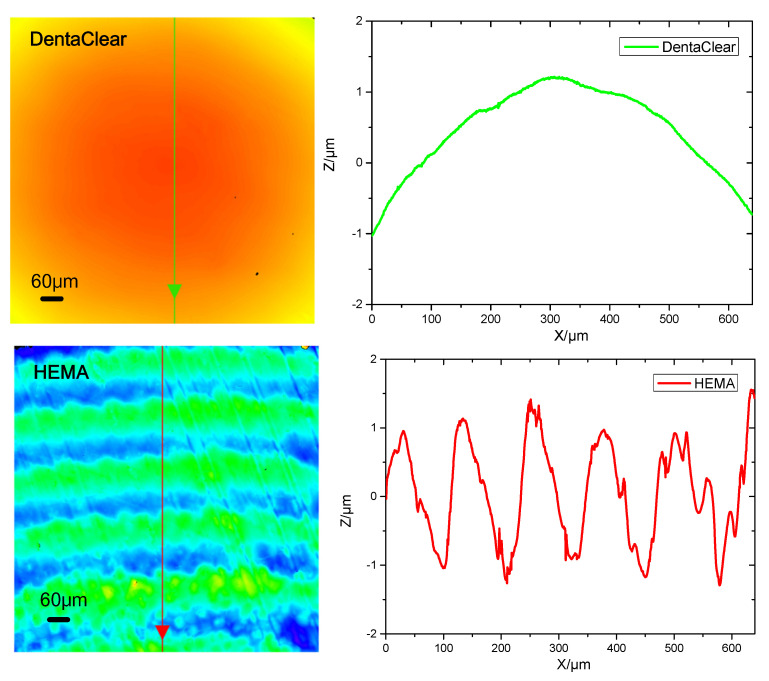
Laser confocal microscope images of the smoothed lens surface and sampling profiles on the surface. The image size is 630 × 630 μm. Apparent ripples appeared on the HEMA surface even if it was smoothed by post-curing, whilst the surface of DentaClear seemed relatively smooth.

The surfaces were also characterized with a laser confocal scanning microscope (Olympus LEXT OLS5100, Tokyo, Japan). The results of the surfaces after smoothing are presented in Figure 7. Similar to the optical microscope results, the stair-stepping effect on the DentaClear lens surface had been greatly mitigated after post-curing smoothing. The surface of DentaClear is quite smooth and the steps cannot be distinguished. The surface roughness is RMS 0.10 μm. In contrast, the stair-stepping effect on the HEMA lens surface remained. The amplitude of ripples on the HEMA lens lies mostly within ~±1 μm with a period ~100 μm, which results in a surface roughness of RMS 0.90 μm. 

**Figure 8:**
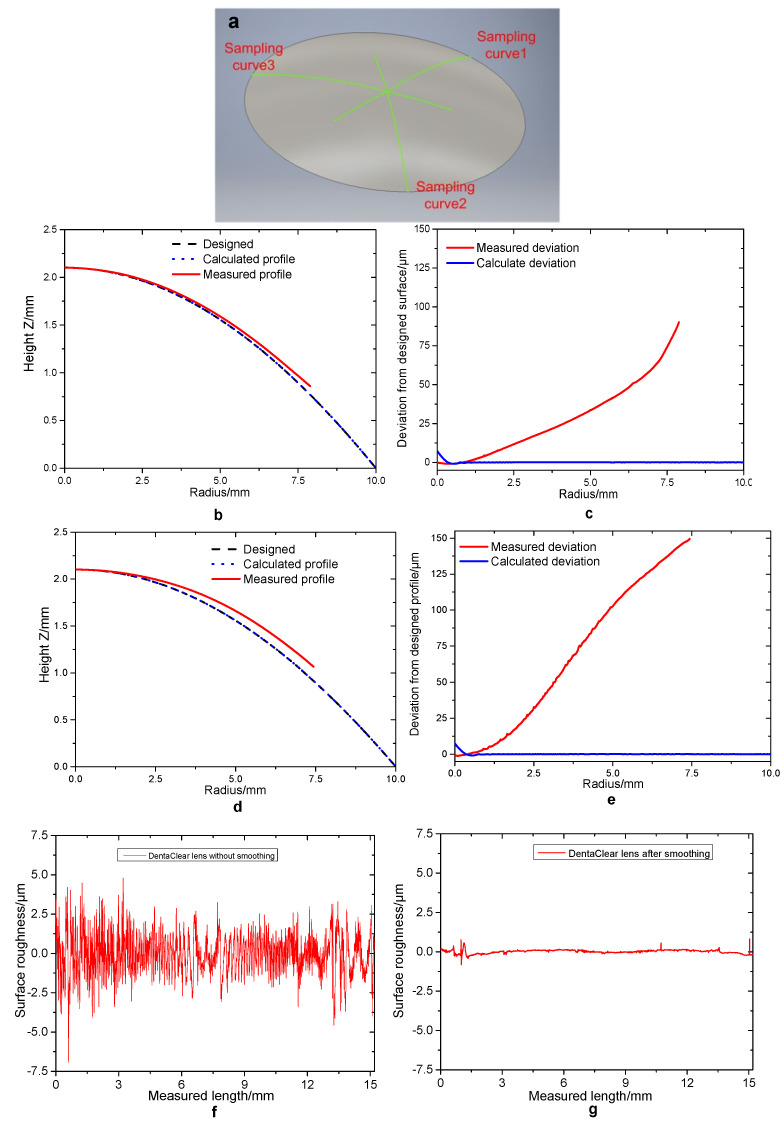

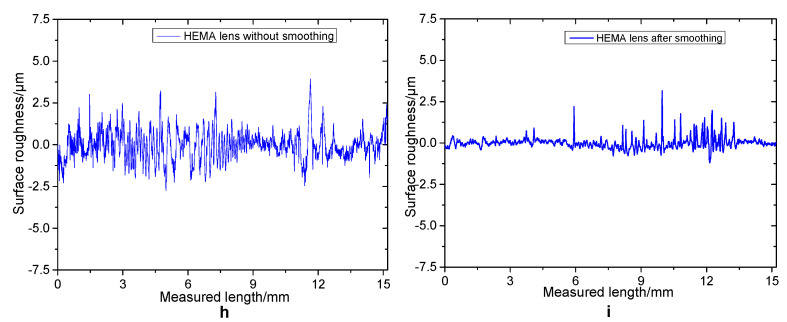
(**a**) Schematic diagram of sampling curves on lens surfaces in the profilometer measurements: each curve has a length of 15.2 mm. (**b**) Printed surface profile and (**c**) deviation of DentaClear lenses compared to the designed profile. (**d**) Printed surface profile and (**e**) deviation of HEMA lenses compared to the designed profile. (**f**) Surface roughness of printed DentaClear lens without smoothing. (**g**) Surface roughness of printed DentaClear lens after smoothing. (**h**) Surface roughness of printed HEMA lens without smoothing. (**i**) Surface roughness of printed HEMA lens after smoothing.

The surface roughness and surface form of the lenses were further measured using stylus profilometry (Form Talysurf PGI 1250S, Taylor Hobson, Leicester, UK). The stylus was a conisphere diamond tip with a 2 μm radius. Figure 8a shows the schematic diagram of sampling curves from this measurement. Three lines of 15.2 mm long passing through the lens vertex were measured. The trace of 15.2 mm is profiled so that the apex of the lens can be readily positioned. The average of three measured surface forms of the smoothed lenses together with the designed surface are plotted in Figure 8b–e. The surface profile deviation of the DentaClear lens was ~74 μm at the radius of 7.5 mm, and for the HEMA lens, the deviation was ~150 μm, about 2 times that of DentaClear. Figure 8f–i shows the surface roughness of the printed lenses before and after smoothing. The surface roughness dropped down from 1.34 and 0.93 μm to 0.16 and 0.43 μm for DentaClear and HEMA after smoothing, a reduction of 88% and 54%, respectively. Table 1 summarizes the surface properties of the printed lenses. It is clear that meniscus equilibrium post-curing can ameliorate the stair-stepping effect and significantly improve the surface roughness of the printed lenses. The smoothing effect, however, was less effective for the HEMA lens.

**Figure 9:**
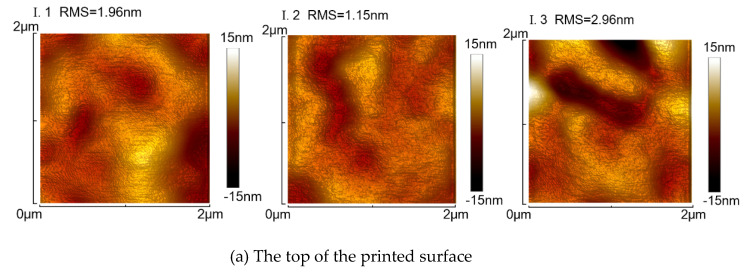

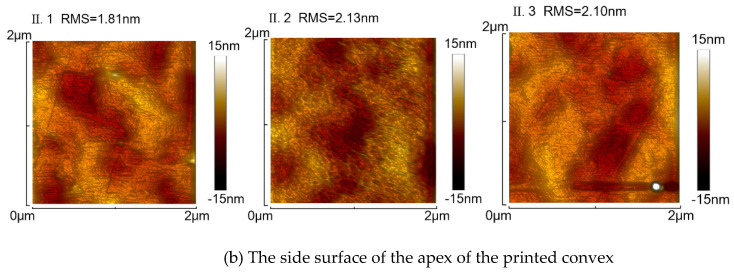
AFM measured surface roughness (2 × 2 μm sampling) on the smoothed DentaClear lens: (**a**) sampling area I on the top surface and (**b**) sampling area II on the side surface.

Moreover, the surface roughness of the smoothed DentaClear lens was further tested using AFM (UPTI-150, Bruker, San Jose, CA, USA). Referring to Figure 6a, sampling area I on the top surface and sampling area II on the side surface, within each sampling area, three 2 × 2 μm regions were scanned, and the morphologies are listed in Figure 9. In sampling area I, the RMS roughness of regions I.1, I.2, and I.3 was 1.96, 1.15, and 1.96 nm, respectively. In sampling area II, the RMS roughness of regions II.1, II.2, and II.3 was 1.81, 2.13, and 2.10 nm, respectively. No significant difference was identified for these two areas. The average surface roughness of all the measured regions was 2.02 nm.

### 4.2. Imaging Performance

**Figure 10:**
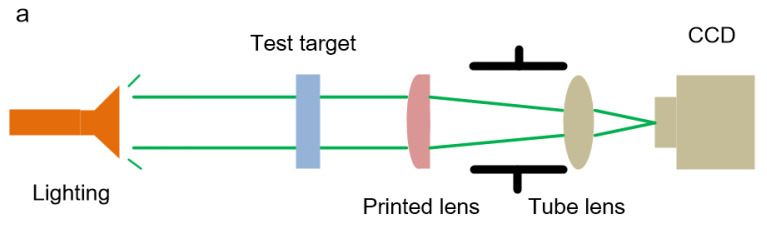

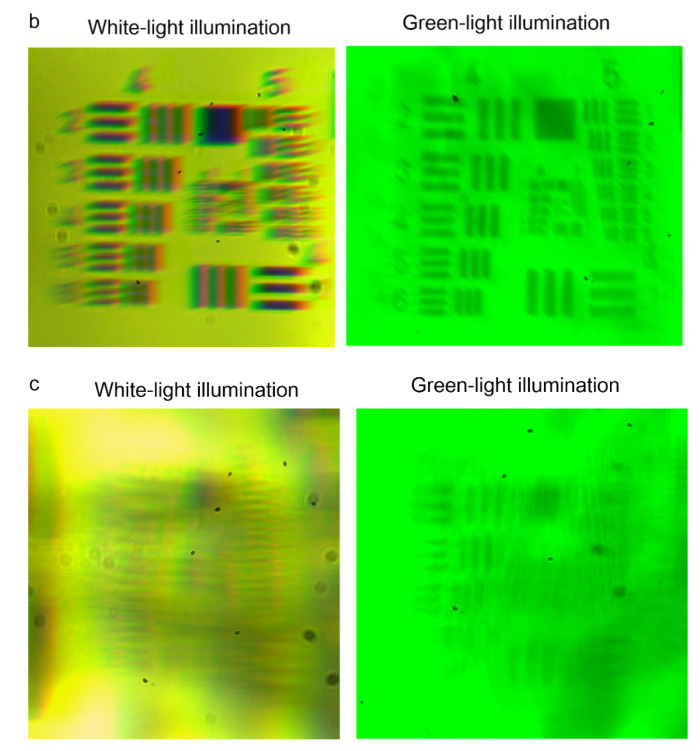
(**a**) Diagrammatic sketch of the experimental setup. (**b**) Recorded images of the DentaClear lens of the resolution target (groups 4–5) with image resolution of 28.50 lp/mm, i.e., res = 8.77 μm, under white-light illumination, 50.80 lp/mm, i.e., res = 4.92 μm, under green-light illumination. (**c**) Recorded images of the HEMA lens of the resolution target (groups 4–5).

As shown in Figure 10a, the imaging performance of the printed lenses was evaluated with the USAF 1951 resolution target (Edmund Optics, Barrington, NJ, USA) at different illumination wavelengths. For the DentaClear lens, the ability of resolving element 5 of group 5 of the test target was demonstrated upon the irradiance of green light (Figure 10b), indicating imaging resolution of 50.80 lp/mm, i.e., res = 4.92 μm. Under white-light irradiation, the image was blurred due to the chromatic aberration, exhibiting an image resolution of 28.50 lp/mm, i.e., res = 8.77 μm. For the HEMA lens, no sharp image was observed in groups 4–5 under either white-light or green-light illumination (Figure 10c). The surface and optical properties of the printed lenses are tabulated in Table 2. The HEMA lens is inferior to the DentaClear lens and shows poor imaging resolution due to low surface quality. The composition of HEMA resin may be the reason for the relatively poor surface quality and optical performance due to the fact that HEMA is much more difficult to cure compared to DentaClear based on the curing thickness experimental results. In addition, HEMA is a kind of acrylic resin, and the curing process is strongly inhibited by oxygen. Therefore, the meniscus layer of HEMA cannot be fully cured, resulting in a poor smoothing effect. The composition should be optimized to improve the hardness of the cured part to reduce the surface profile deviation, the UV-absorber could be replaced by a more efficient one, and the meniscus layer for smoothing may be cured in a vacuum environment.

## 5. Conclusions

We have described a process for fabricating customized aspheric lenses, in which a stereolithographic 3D printer is combined with the meniscus equilibrium post-curing technique. The effectiveness of the meniscus equilibrium post-curing for surface smoothing was theoretically analyzed, and the results show that the deviations between calculated profiles and the designed profiles range from −0.89 to around 7.48 μm for both resins. 

Post-curing can smooth the printed lenses of both DentaClear and HEMA, and appeared to be more effective for DentaClear. The profile deviations for DentaClear and HEMA from the designed profile were ~74 and ~150 μm, respectively. The surface roughness was also evaluated by means of stylus profiler, AFM, and LCSM. The surface roughness of DentaClear (0.16 μm) was superior to that of HEMA (0.43 μm). The printed DentaClear lens can resolve a target of as fine as 50.80 lp/mm, i.e., res = 4.92 μm, under the illumination of green light.

In summary, we have demonstrated that stereolithography 3D printing combined with the meniscus equilibrium post-curing technique is a simple and effective approach to create customized aspheric lenses. The commercial DentaClear lens is of low surface profile deviation and has satisfactory optical imaging resolution. However, the HEMA lens was of rough surface and poor optical performance. Further experiments should be carried out to optimize the resin composition. In addition, the mechanical and optical properties of the printed lens should be further improved for the wide application of 3D-printed lenses. For example, by investigating new formulations of photocurable resins, as reported by Kotz et al. [21] and Moore et al. [22], fused silica components with complex structures can be fabricated using 3D printing technology. Our work illustrates that 3D printing technology is a potential method for creating optical components, which may change the future approach of customized optical manufacturing.

## Figures and Tables

**Table 1:** Surface properties of the lenses without and after smoothing.

	DentaClear lens			HEMA lens		
	Stair-Stepping Effect	Surface Roughness Measured by Profile Meter	Deviation from Designed Profile	Stair-Stepping Effect	Surface Roughness Measured by Profile Meter	Deviation from Designed Profile
Before smoothing	visible	1.34 μm	N/A	visible	0.93 μm	N/A
After smoothing	invisible	0.16 μm	~74 μm at 7.5 mm	visible	0.43 μm	~150 μm at 7.5 mm

**Table 2:** Optical properties of the fabricated lenses.

	Surface Properties		Image Resolution	
	Surface Roughness Measured by Stylus Profilometry	Surface Roughness Measured by AFM	White Light	Green Light
DentaClear lens	0.16 μm	2.02 nm	8.77 μm	4.92 μm
HEMA lens	0.43 μm	N/A	N/A	N/A

## Data Availability

The data presented in this study are available on request from the corresponding author.

## References

[1] Fattoum E.Y., Al-Khateb E.Y., Katnah A., Jabra R. (2017). Design, manufacturing and measurement of aspheric test-plate using only traditional techniques. J. Opt..

[2] Chen X., Liu W., Dong B., Lee J., Ware H.O.T., Zhang H.F., Sun C. (2018). High-speed 3D printing of millimeter-size customized aspheric imaging lenses with sub 7 nm surface roughness. Adv. Mater..

[3] Thiele S., Arzenbacher K., Gissibl T., Giessen H., Herkommer A.M. (2017). 3D-printed eagle eye: Compound microlens system for foveated imaging. Sci. Adv..

[4] Van Lith R., Baker E., Ware H., Yang J., Farsheed A.C., Sun C., Ameer G. (2016). 3D-printing strong high-resolution antioxidant bioresorbable vascular stents. Adv. Mater. Technol..

[5] Kotz F., Risch P., Helmer D., Rapp B.E. (2018). Highly fluorinated methacrylates for optical 3D printing of microfluidic devices. Micromachines.

[6] Eckel Z.C., Zhou C., Martin J.H., Jacobsen A.J., Carter W.B., Schaedler T.A. (2016). Additive manufacturing of polymer-derived ceramics. Science.

[7] Surdo S., Carzino R., Diaspro A., Duocastella M. (2018). Single-Shot Laser Additive Manufacturing of High Fill-Factor Microlens Arrays. Adv. Opt. Mater..

[8] Xing J., Rong W., Sun D., Wang L., Sun L. (2016). Extrusion printing for fabrication of spherical and cylindrical microlens arrays. Appl. Opt..

[9] Gissibl T., Thiele S., Herkommer A., Giessen H. (2016). Two-photon direct laser writing of ultracompact multi-lens objectives. Nat. Photonics.

[10] Assefa B.G., Saastamoinen T., Biskop J., Kuittinen M., Turunen J., Saarinen J. (2018). 3D printed plano-freeform optics for non-coherent discontinuous beam shaping. Opt. Rev..

[11] Shao G., Hai R., Sun C. (2020). 3D printing customized optical lens in minutes. Adv. Opt. Mater..

[12] Assefa B.G., Pekkarinen M., Partanen H., Biskop J., Turunen J., Saarinen J. (2019). Imaging-quality 3D-printed centimeter-scale lens. Opt. Express.

[13] Melchels F.P.W., Feijen J., Grijpma D.W. (2010). A review on stereolithography and its applications in biomedical engineering. Biomaterials.

[14] Berglund G.D., Tkaczyk T.S. (2019). Fabrication of optical components using a consumer-grade lithographic printer. Opt. Express.

[15] Vaidya N., Solgaard O. (2018). 3D printed optics with nanometer scale surface roughness. Microsyst. Nanoeng..

[16] Pan Y., Zhao X., Zhou C., Chen Y. (2012). Smooth surface fabrication in mask projection based stereolithography. J. Manuf. Process..

[17] Pan Y., Chen Y. (2016). Meniscus process optimization for smooth surface fabrication in Stereolithography. Addit. Manuf..

[18] Pan Y., Chen Y. (2015). Smooth surface fabrication based on controlled meniscus and cure depth in microstereolithography. J. Micro Nano-Manuf..

[19] Raman R., Bhaduri B., Mir M., Shkumatov A., Lee M.K., Popescu G., Kong H., Bashir R. (2016). High-resolution projection microstereolithography for patterning of neovasculature. Adv. Healthcare Mater..

[20] Zmarzły P., Gogolewski D., Kozior T. (2020). Design guidelines for plastic casting using 3D printing. J. Eng. Fibers Fabr..

[21] Kotz F., Arnold K., Bauer W., Schild D., Keller N., Sachsenheimer K., Nargang T.M., Richter C., Helmer D., Rapp B.E. (2017). Three-dimensional printing of transparent fused silica glass. Nature.

[22] Moore D.G., Barbera L., Masania K., Studart A.R. (2020). Three-dimensional printing of multicomponent glasses using phase-separating resins. Nat. Mater..

